# Micro-Computed Tomography beamline at the Australian Synchrotron: data acquisition and processing pipeline

**DOI:** 10.1107/S160057752600130X

**Published:** 2026-03-11

**Authors:** Darren Thompson, Benedicta D. Arhatari, Andrew Stevenson

**Affiliations:** a Australian Synchrotron, ANSTO, Clayton, Victoria3168, Australia; RIKEN SPring-8 Center, Japan

**Keywords:** X-ray tomography, synchrotron, data acquisition, processing pipeline

## Abstract

The Micro-Computed Tomography (MCT) beamline at the Australian Synchrotron (ANSTO) has undergone significant advancements in data acquisition and processing infrastructure to support the growing diversity of user needs and experimental techniques. Originally designed for conventional X-ray computed tomography, the beamline now accommodates advanced imaging modalities such as speckle-based and grid-based phase contrast imaging. These improvements were made possible by combining fast data transfer, powerful computing with GPUs, and large data storage. A flexible and user-friendly software system is helping researchers control experiments and process data efficiently. The result is a fast, reliable, and adaptable system that meets the needs of a wide variety of users, requiring many different experimental configurations.

## Abstract 2

The Micro-Computed Tomography (MCT) beamline at the Australian Synchrotron has undergone significant advancements in data acquisition and processing infrastructure to support the growing diversity of user needs and experimental techniques. Recent developments in data acquisition and processing infrastructure at the beamline are presented.

## 1. Introduction

X-ray computed tomography (CT) experiments conducted at synchrotron radiation facilities generate large and complex datasets, including both raw acquisition data and high-resolution reconstructed volumetric images. To accommodate the wide range of user experience levels (Charles *et al.*, 2024 ▸; Blake *et al.*, 2024 ▸; McArdle *et al.*, 2024 ▸), the data acquisition system must be not only robust but also intuitive and easy to operate (Brun *et al.*, 2015 ▸; Brun *et al.*, 2017 ▸). The high data throughput associated with synchrotron CT necessitates advanced computing infrastructure for efficient data handling and processing (Kachelriess *et al.*, 2008 ▸; Marone *et al.*, 2017 ▸; Thompson *et al.*, 2011 ▸). This includes high-performance computing clusters, GPU-accelerated pipelines, and scalable storage architectures capable of managing petabyte-scale data volumes. Furthermore, the diverse scientific requirements of beamline users, which may vary from real-time image reconstruction to advanced post-processing and analysis, demand flexible, customizable solutions (Hintermüller *et al.*, 2010 ▸). The ability to scan a broad range of sample types is essential, particularly given the varied research backgrounds and imaging goals of the user community. These solutions must support heterogeneous computational workflows, ensure data integrity, and facilitate seamless data access, sharing, and long-term archiving. Optimizing the computational and storage ecosystem (De Carlo *et al.*, 2014 ▸) is therefore critical to maximizing the scientific output of CT experiments at synchrotron facilities (Goscinski *et al.*, 2014 ▸).

At the Australian Synchrotron, beamline access is provided through merit-based research proposals, preferred access schemes, and commercial contracts. Since commencing user operations in October 2022, the MCT (Micro-Computed Tomography) beamline has hosted 156 experiments (100 via merit access and 56 via preferred access) serving a total of 707 user visits. The MCT beamline is designed to support a wide variety of experimental programs across diverse research disciplines (Mayo *et al.*, 2013 ▸). As summarized in Table 1 ▸, experiments from a range of fields have been conducted at the MCT beamline, with the three most popular fields of research (FoR) identified for each experiment round, with three rounds held each year. Over a three-year period, materials engineering has been the most popular topic, accounting for the largest share of experiments (12%), followed by geology (9%) and classical physics (5%).

To address the challenges of high-throughput CT, the MCT beamline has adopted a multi-layered approach, led by the Scientific Computing team. On the hardware side, a high-speed network infrastructure enables efficient data transfer between acquisition systems, storage servers and computing clusters. Although other experimental hardware such as the monochromator, mirror, motors, photon shutter *etc*. are also critical, it is beyond the scope of this paper. On the software side, the MCT beamline features a modern data acquisition and management architecture, as well as optimized algorithms for fast parallelized data processing. These include Feldkamp–Davis–Kress (FDK) (Vaniqui *et al.*, 2019 ▸), filtered back-projection (FBP) (Kak & Slaney, 1988 ▸) and iterative CT reconstruction methods that leverage GPU acceleration to achieve near real-time performance (Thompson *et al.*, 2011 ▸; Kachelriess *et al.*, 2008 ▸). A data management system with automated indexing, metadata tagging and hierarchical storage management enables efficient data organization and retrieval. The beamline also provides a high-capacity data storage system, currently supporting up to 2 Petabytes (2 × 10^6^ Megabytes), to handle large-scale datasets. Additionally, the MCT processing pipeline runs on ASCI (Australian Synchrotron Computing Infrastructure), a high-performance computing system that enhances scalability and provides remote access capabilities for users (Marcou *et al.*, 2018 ▸).

User-specific customization is another essential feature, as many experiments require adaptation beyond conventional tomography setups (Brun *et al.*, 2015 ▸). This includes advanced imaging modalities such as speckle-based and grid-based phase contrast imaging (Alloo *et al.*, 2025 ▸) or scanning techniques using a patterned probe (Aminzadeh *et al.*, 2025 ▸). Speckle- and grid-based phase-contrast X-ray imaging are techniques used to study materials that weakly absorb X-rays. They provide information on absorption, refraction (phase shift) and small-angle scattering (dark-field). A speckle or grid pattern is placed in the beam to create a reference image, which is slightly distorted when a sample is introduced (Morgan *et al.*, 2011 ▸). Several speckle or grid images are taken both with and without the sample, and the differences between them are analysed to extract absorption, phase and dark-field information. These methods can be used in both tomography and radiography (Zdora, 2018 ▸). To support such diversity, the MCT beamline offers a modular data acquisition system with an intuitive user interface enabling users to design, configure and optimize workflows with minimal complexity. Finally, comprehensive data security protocols including user authentication, encryption and automated backup strategies are implemented to ensure the protection and integrity of experimental data.

The MCT beamline provides white, pink and monochromatic X-ray beams (Arhatari *et al.*, 2023 ▸; see also https://asuserwiki.atlassian.net/wiki/spaces/UO/pages/1516601345/MCT+Beamline). The white beam consists of a filtered polychromatic spectrum from the bending-magnet source. The pink beam is produced by suppressing the high-energy portion of the white beam using a vertical bounce mirror (VBM). Monochromatic radiation is generated using a double-multilayer monochromator (DMM), delivering an energy bandpass of 0.5% or 3% depending on the selected multilayer stripe (Arhatari *et al.*, 2025 ▸). The MCT beamline is equipped with three detectors: white, mono and high speed. Each detector is fitted with a range of objective lenses that can be easily selected to provide the required image magnification, field of view and corresponding spatial resolution for each experiment (Arhatari *et al.*, 2023 ▸). White and mono detectors are coupled to a high-resolution pco.edge 5.5 sCMOS 16-bit camera, featuring 2560 × 2160 pixels with a pixel size of 6.5 µm × 6.5 µm and a maximum frame rate of 100 frames s^−1^. Fast data collection can be achieved either by reducing the number of vertical image rows using the pco.edge detector or by employing a high-speed detector (Phantom S710). The Phantom detector is capable of recording at over 7000 frames s^−1^, with an image size of 1280 × 800 pixels and a pixel size of 20 µm × 20 µm. Although the Phantom S710 offers a higher frame rate, its larger pixel size is a drawback for some applications. In fact, practical frame rates achieved are usually limited by X-ray flux. The data-acquisition system is based on *EPICSareaDetector*, which is used to control the cameras. However, data are not written directly to disk using the standard *areaDetector* HDF5 plugin. Instead, an in-house-developed *areaDetector* plugin (*ADPluginRedis*) is used to stream image data into a *Redis* in-memory database. This design decouples acquisition from disk I/O and allows data to be written to disk asynchronously by a separate process, thereby avoiding I/O-related performance limitations during high-speed acquisition. The HDF5 file is created and written by the acquisition aggregator service which combines data from *areaDetector* data extracted from *Redis* and data extracted from the *Bluesky* document stream.

The primary aim of this paper is to present recent developments in data acquisition and processing infrastructure at the MCT beamline. While the beamline originally focused on supporting conventional CT experiments, ongoing improvements have expanded its capabilities significantly. Through the integration of advanced software tools, flexible hardware configurations, and scalable processing pipelines, the MCT beamline now serves as a versatile platform capable of supporting a wide range of scientific imaging techniques and user needs.

## 2. Data acquisition

**Figure 1:**
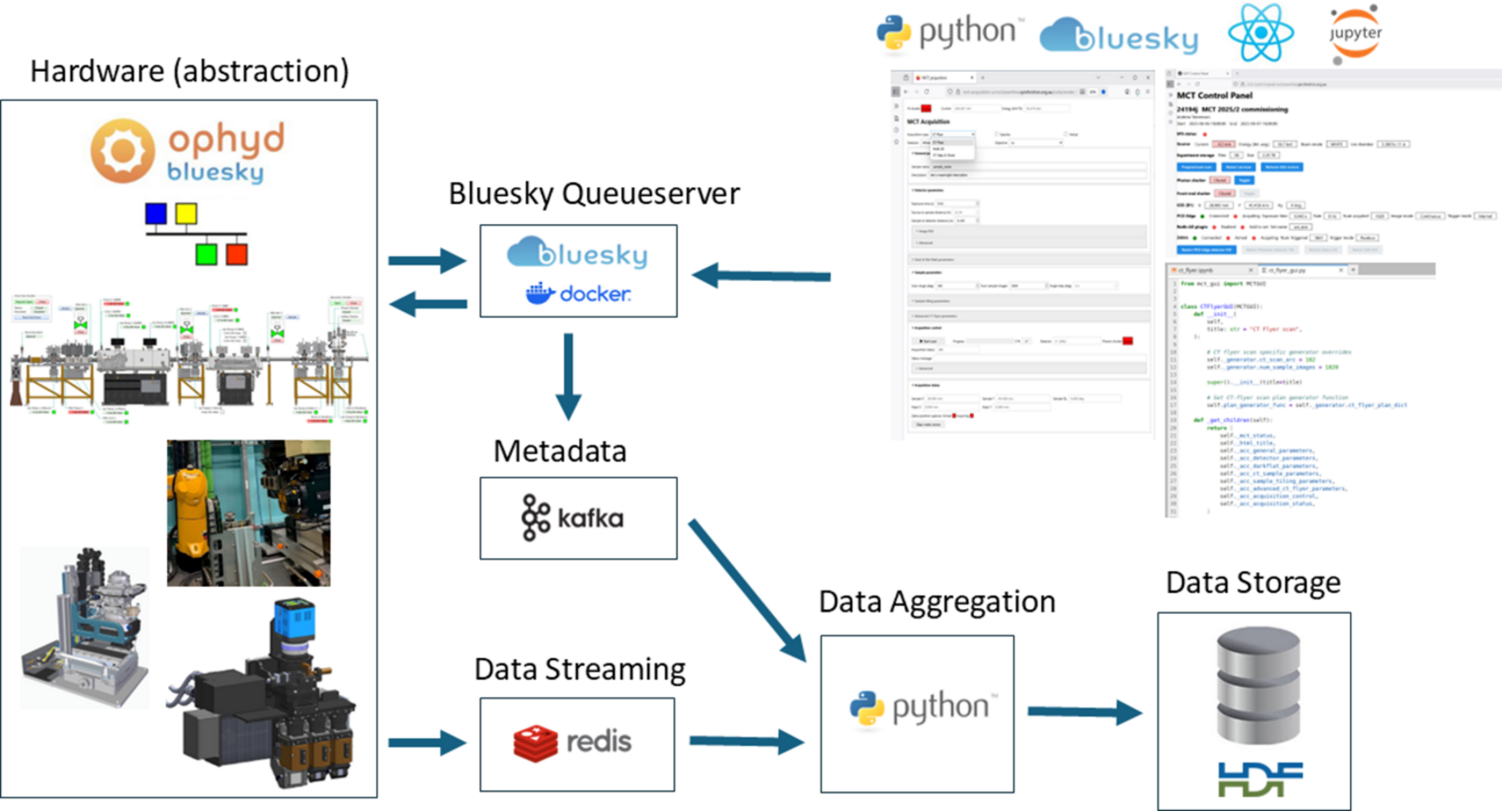
High-level overview of a data acquisition and management architecture at the MCT beamline.

The MCT data acquisition system was developed in-house by the Scientific Computing group at the Australian Synchrotron, using a mixture of existing open-source and custom written tools. Fig. 1 ▸ presents a schematic of the data acquisition, management and processing ecosystem as implemented at the MCT beamline.

The MCT acquisition system consists of several software micro-services working in unison and deployed on a containerized Kubernetes cluster (https://kubernetes.io/docs/concepts/containers/images/). At the core of the system is the abstraction of hardware *EPICS*-based devices using the Python *Ophyd* library (https://blueskyproject.io/ophyd/user_v2/). These device definitions are stored in the *mct-beamline-library*. *Ophyd* device wrappers are created for components such as monochromator, motion stages, filters, detectors, Zebra device, *etc*. enabling experimental hardware to be controlled and coordinated programmatically. These *Ophyd* devices are managed using *Bluesky* plans (Allan *et al.*, 2019 ▸), which orchestrate specific tasks such as tomography or multi-2D acquisitions, allowing seamless automation of image capture from detectors. The *Bluesky Run Engine*, deployed as part of a *Bluesky*-*Queueserver* instance within a containerized Docker environment, oversees experiment execution. For users, experimental acquisitions on the MCT beamline are specified and initiated via the MCT data acquisition web-based GUI (graphical user interface). The GUI permits acquisition parameters such as the file name, exposure time, camera-objective magnification, number of projections, number of dark- and flat-field images *etc*. to be defined. This design enables users to create, execute and monitor experiments in real time using flexible, scriptable and well documented tools. All data entered or captured during an MCT acquisition, both images, experimental data and metadata are stored in a single Hierarchical Data Format (HDF5) file (https://www.hdfgroup.org). This format is particularly effective for managing large, complex scientific datasets.

A file skeleton is created at the beginning of each acquisition plan, executed by the *Bluesky*-*Queueserver*. This creates the overall structure of image datasets, groups and attributes to be populated during the main acquisition phases. A significant proportion of experimental information is known prior to acquisition, and such data are pre-written during this phase. This includes fixed and user-specified parameters such as image volume sizes *etc*. This design allows for the corresponding acquisition HDF5 file to be ‘filled in’ by the acquisition ‘aggregator’ micro-service both during and after the acquisition completion.

Non-image experimental data and metadata produced during experiments, including scan parameters, timestamps, motor positions and device configurations, which are captured by the *Bluesky* document stream, are subsequently re-routed into a *Kafka* (https://kafka.apache.org/) stream, a real-time streaming platform that facilitates traceability and integration with external systems. Simultaneously, compressed raw detector data are streamed to temporary in-memory storage utilizing the high-performance *Redis* platform (https://redis.io/open-source/) via an in-house-developed *EPICSareaDetector* plugin (*ADPluginRedis*). Tomographic projections are generally information dense and usually do not exhibit good compression from standard compression algorithms in the time available. However, Blosc compression is enabled via the standard *areaDetectorNDPluginCodec*; this plugin is used in series as an input to the downstream *ADPluginRedis* plugin providing compressed frames which are then sent for temporary storage in *Redis* prior to writing to the MCT acquisition HDF5 file by the aggregator service. Both plugins are configured to run in multi-threaded mode. The *Codec* plugin is configured with bitshuffle enabled, ZSTD compression and five Blosc threads by default. By comparison, the *Redis* plugin is configured to run via four threads. With this configuration, projections are generally compressed by between 5% and 10% with minimal CPU load, even when running at high speed (>500 frames s^−1^). It should be noted that projections are subsequently decompressed when extracted from *Redis* by the aggregator and are written as uncompressed 3D datasets in the HDF5 file. *Redis* storage provides a flexible and effective ‘frame-buffering’ mechanism, permitting physical file-I/O (Input Output) to be fully disconnected and asynchronous from the acquisition phase, freeing it from performance and timing constraints. *Redis* also provides a rich set of features for checking and validation of data. Some NDAttributes are utilized from decompressed projections extracted from *Redis*, mainly for some basic checks and validation by the acquisition aggregator service. However, most metadata are extracted and persisted into the HDF5 file from *Ophyd* device metadata extracted from the *Bluesky* document stream. As we are not using an AD file writing plugin, per projection metadata handling is done differently. Finally, during an acquisition, the *mct-acquisition-aggregator* micro-service monitors and extracts data from both *Redis* and the *BlueskyKafka* document stream populating the HDF5 file with experimental data. Metadata are contained in the HDF5 file and available for users for both raw files and for the reconstructed file.

In synchrotron tomography, the sample is placed in the X-ray beam path between the source and the imaging detector. During data acquisition, the sample is rotated through a 180° range while the detector captures a series of X-ray intensity images. To correct for inhomogeneities in the X-ray beam and imperfections in the detector system, additional reference images are required. These include flat-field images (acquired without the sample, to correct for beam profile and imperfections in the detector system) and dark-field images (acquired without X-rays, to correct for detector noise). These reference images are typically collected before and/or after each tomography scan. The MCT data acquisition web GUI provides options for capturing these images. These three datasets—projections, flat fields and dark fields—along with rotation angle metadata, form the input to the MCT processing pipeline.

**Figure 2:**
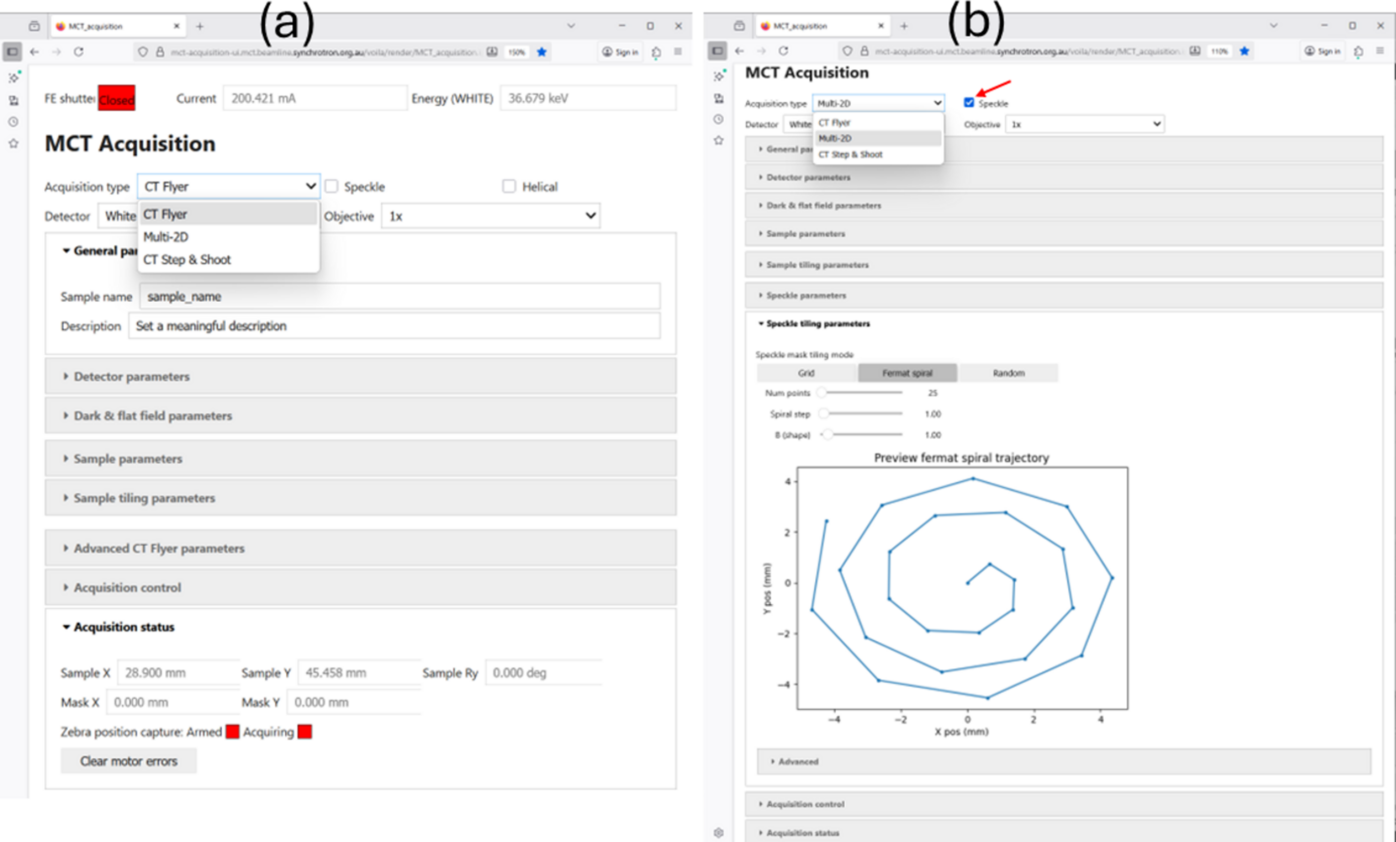
MCT data acquisition web GUI for controlling (*a*) CT flyer scans and (*b*) multi-2D speckle scans. Users can easily set scan parameters, enabling precise and flexible experiment control.

The MCT data acquisition web GUI supports a range of different experimental modalities, tailored for both advanced and general users. Supported modalities include CT flyer scan, CT step-and-shoot and a multi-2D scan, which can be selected from the drop-down list in the GUI as shown in Fig. 2 ▸(*a*). Furthermore, speckle-based imaging can be enabled for each mode, and a helical mode can be specified for CT based modes. For tomographic scanning, two modes are available: flyer and step-and-shoot scans. In CT flyer scan mode, the sample rotates continuously while the detector captures projection data. A ‘Zebra’ device (https://quantumdetectors.com/zebra/)—a digital signal level converter and position capture unit—coordinates triggering between the rotation stage and the detector. The experimental parameters for flyer scans are built into the software, so the user only needs to specify the exposure time, and the system automatically adjusts the rotation speed of the stage accordingly. In contrast, step-and-shoot mode involves stopping the rotation stage at each projection angle for image acquisition.

Fig. 2 ▸ shows parameters organized into related collapsible sections (*e.g.* Detector, Sample, Tiling and Acquisition control); several sections include additional Advanced sub-sections containing advanced and less commonly used parameters. The GUI displays live information such as front-end shutter and beamline shutter status, beam current, X-ray energy and sample position, enhancing situational awareness during scans. Moreover, the GUI includes scan progress tracking, status messages and hardware state (*e.g.* Zebra arming and detector activity), supporting robust and transparent acquisition management.

Below summarizes the key steps involved in performing a CT flyer scan at the MCT beamline:

(1) Collect pre-scan flat and dark images (involving control of linear translation stage and beamline shutters, respectively).

(2) Move the rotation stage to 0° and recalibrate Zebra to define the zero position.

(3) Move the stage to a negative start angle (based on the calculated speed) to allow ramp-up time.

(4) Configure Zebra to begin triggering at 0°, generating pulses at the required angular interval. MCT CT flyer supports both time and position-based triggering.

(5) Set the detector to external triggering mode.

(6) As the stage reaches 0°, Zebra starts triggering the detector and records position data.

(7) Continue rotating the stage beyond the scan range.

(8) Once the desired number of images has been collected and propagated through the *Redis* plugin:

 (*a*) Stop the rotation stage.

 (*b*) Stop detector acquisition.

 (*c*) Reset Zebra to end data capture.

 (*d*) Trim any excess position data to match the number of acquired images.

 (*e*) Return the stage to 0° and reset devices.

For scanning samples larger than the field of view (FOV), the MCT acquisition GUI supports tiled scanning, allowing scans in both horizontal and vertical directions using a grid-based motion path. For tiled scanning, the sample tiling parameters (the number of tiles and step size) are determined during the alignment procedure before data collection. These parameters are identified using the motor travel distances in the Engineering GUI together with the live detector view in the Detector GUI. These values are then entered into the web-based acquisition GUI as the sample tiling parameters. For speckle or grid-based imaging experiments, additional GUI sections are provided for tomography and radiography speckle scans. These experiments introduce an extra stage to hold the speckle or grid pattern (Alloo *et al.*, 2025 ▸; Aminzadeh *et al.*, 2025 ▸). The speckle-based GUI enables control over this additional mechanical stage, supporting both standard grid trajectories and non-grid patterns such as random or Fermat spiral paths (Huang *et al.*, 2014 ▸). An example of the multi-2D speckle GUI with Fermat spiral trajectory selected is shown in Fig. 2 ▸(*b*). Users can easily set the number of points, spiral step, and shape, allowing flexibility in conducting the experiment. It should be noted that, in the case of tiled and speckle scans where multiple sets of dark, flat and sample images are acquired, all data are still aggregated and contained in a single HDF5 file and are considered to be a single acquisition in this context.

## 3. Processing pipeline

The MCT processing pipeline is accessible through the ASCI (Australian Synchrotron Computer Infrastructure) desktop, which provides a web-based Linux environment for data processing (Marcou *et al.*, 2018 ▸). Experimental data are directly available within the ASCI desktop. During beam time, users access an ‘MCT Online’ session with dedicated high-performance computing resources for faster processing. After beam time, users can continue their work using the ‘MCT Offline’ session, which is accessible remotely outside the facility and offers the same software and capabilities on shared computing infrastructure. Analysis software such as *ORS-Dragonfly*, *3D Slicer* and *FIJI* are also available on the ASCI desktop for MCT users. However, *Dragonfly* is only available for online users. These tools support a wide range of post-processing tasks, including 3D visualization, image rendering, segmentation for volume calculation, pore network analysis, bone structure analysis, and more, making them valuable for both qualitative and quantitative image analysis.

**Figure 3:**
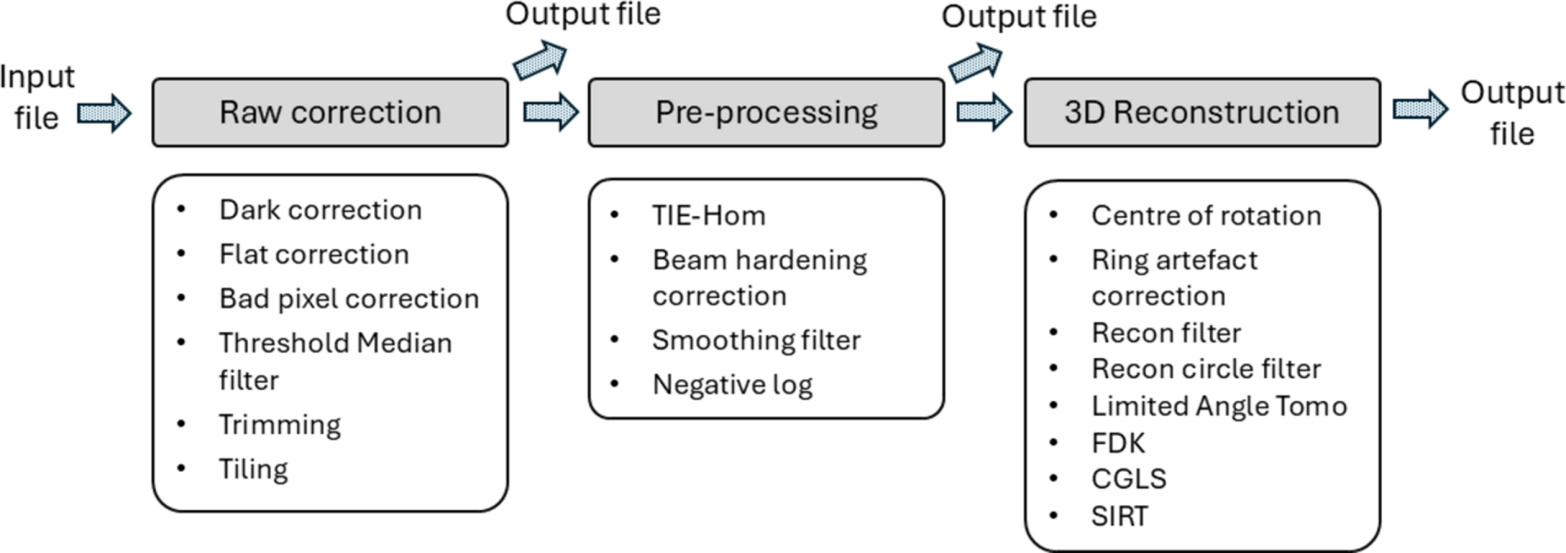
The MCT reconstruction pipeline consists of raw correction, pre-processing and reconstruction sections. Each section contains several features and can generate output files if required.

**Figure 4:**
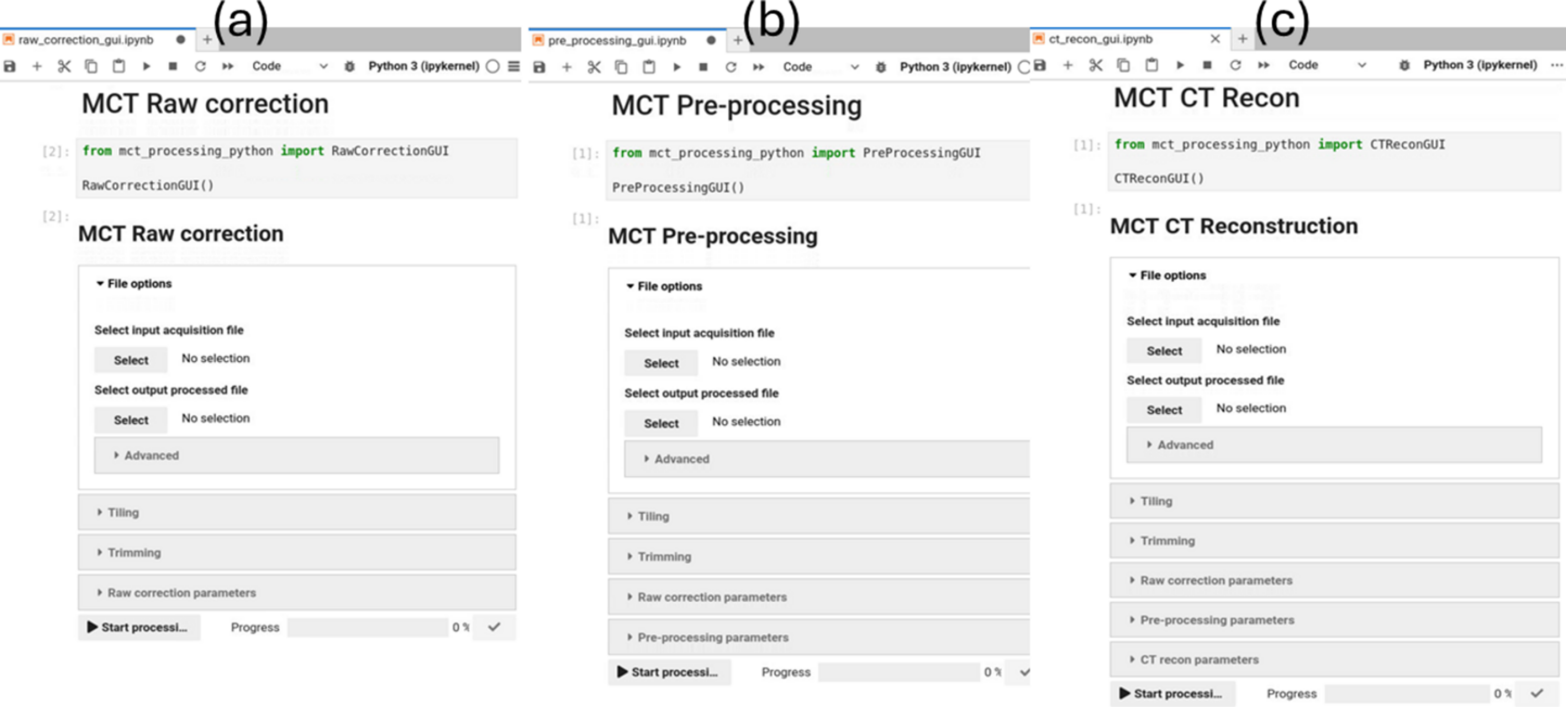
MCT processing of the (*a*) raw correction GUI, (*b*) pre-processing GUI and (*c*) CT reconstruction GUI, available for users in ASCI high-performance computing desktop.

The MCT processing pipeline, developed in-house by the Scientific Computing group, is divided into three main sections: raw correction, pre-processing and 3D reconstruction, as illustrated in Fig. 3 ▸. Each projection image undergoes raw correction and/or pre-processing before 3D reconstruction, and each section can generate output files if required. The pipeline is built on the open-source *Insight Toolkit* (*ITK*) (McCormick *et al.*, 2014 ▸) and incorporates *ASTRA Toolbox* (van Aarle *et al.*, 2016 ▸), all managed through a *Jupyter Notebook* interface that leverages the custom-developed algorithms. Python wrappers based on *ITK* are available for all elements of the MCT pipeline, enabling it to be configured, executed and modified directly using the Python programming language. The *Jupyter Notebook*, specifically designed for processing MCT HDF5 files, runs within a custom-built containerized environment on the ASCI platform, pre-packaged with all necessary software and dependencies. Most of the pipeline’s code is custom written as *ITK* modules in C++ (CUDA), optimized for GPU acceleration, supporting multi-GPU usage, and accessed through specialized Python wrappers. To maximize performance, the pipeline takes full advantage of advanced CUDA capabilities (Vingelmann & Fitzek, 2020 ▸), including streaming, pinned/unified memory, asynchronous memory transfers and kernel launches. The processing pipeline GUIs (as shown in Fig. 4 ▸) operate on a high-performance machine (ASCI) during experiments, equipped with 2 TB of RAM, 256 CPU cores and three high-spec GPUs for ASCI Online, and 1 TB of RAM, 128 CPU cores and one high-spec GPU for ASCI Offline. Although the system provides substantial storage capacity, its transfer performance can be somewhat limited.

Output from the processing pipeline can be written in several data formats beyond the default HDF5 format. Available options include TIFF, TIFF Stack, MDA, VTK, DICOM, NRRD and NIFTI. Among these, NIFTI and DICOM are the most universal and well integrated formats, allowing seamless import into analysis software such as *Dragonfly*, *Avizo* and *3D Slicer* without the need for manual adjustments to pixel spacing or other metadata.

### 3.1. Raw correction

Raw correction is the first stage of the processing pipeline, as shown in Fig. 3 ▸. Once the data and corresponding projection angles are imported, the raw correction module provides a set of default methods to prepare the data for reconstruction. This process begins with *dark and flat correction*, where the intensity projection, *I*_cor_, is adjusted using the following formula,
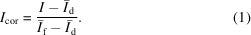
Here, *I* represents the raw intensity, 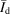 is the averaged dark intensity and 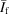 is the averaged flat intensity.

**Figure 5:**
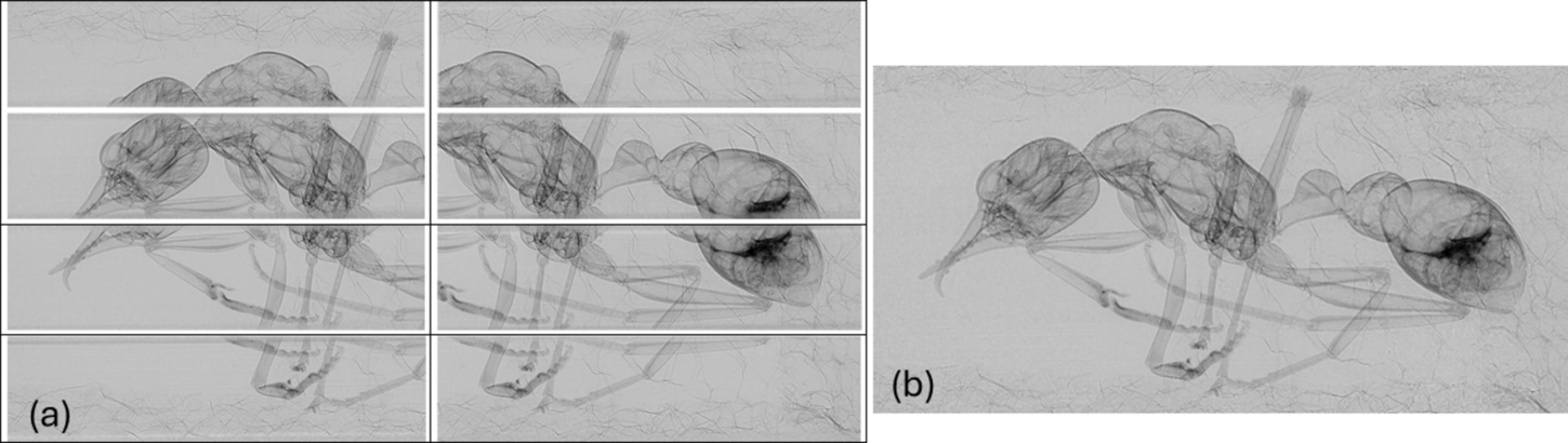
Projection image of an ant sample (2.4 cm in length) after raw correction section shown (*a*) in a 2×4 tiling acquisition layout and (*b*) after the tiling procedure has been applied.

By default, *bad pixel correction* is enabled, while a *threshold median filter* can be toggled on or off as needed. The *trimming* step allows users to crop the projection images or reduce the number of projections. This is particularly useful when processing projection data alone, such as when viewing tiled or stitched projection images. The *tiling* step is automatically applied to tiled data and supports two methods: dead reckoning and correlation. Dead reckoning, which calculates tile positions based on motor position, is typically effective for low-magnification datasets. In contrast, high-magnification datasets benefit from the correlation method, which optimally aligns sub-scan tiles for precise stitching. Correlation based stitching is implemented using the *ITK* Montage module (Zukić *et al.*, 2021 ▸). Fig. 5 ▸ shows an example of a projection image of an ant before and after the tiling procedure in the raw correction stage. An overlapping region between adjacent sub-sections is essential in setting the stitching parameters in order for the tiling algorithm to effectively process the data. Users should be mindful that stitching in the horizontal and/or vertical direction can significantly increase the size of the reconstructed data.

### 3.2. Pre-processing

Pre-processing is the second stage of the pipeline, providing several optional operations (see Fig. 3 ▸). One key feature of this stage is the *TIE-Hom-based phase-retrieval* algorithm (Paganin *et al.*, 2002 ▸), which extracts phase information from the projected intensity dataset, as follows,
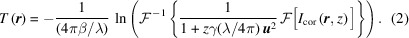
This algorithm requires only a single propagation distance, *z*, and the parameter γ (the ratio δ/β from the X-ray complex refractive index of the material), where δ relates to phase shift and β relates to attenuation. The γ value is determined by the X-ray energy (or X-ray wavelength, λ) used in the experiment. Here 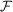 is the Fourier transform operator with respect to the transverse coordinates ***r***, 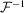 is the corresponding inverse Fourier transform, ***u*** is the Fourier variable conjugate to ***r***.

**Figure 6:**
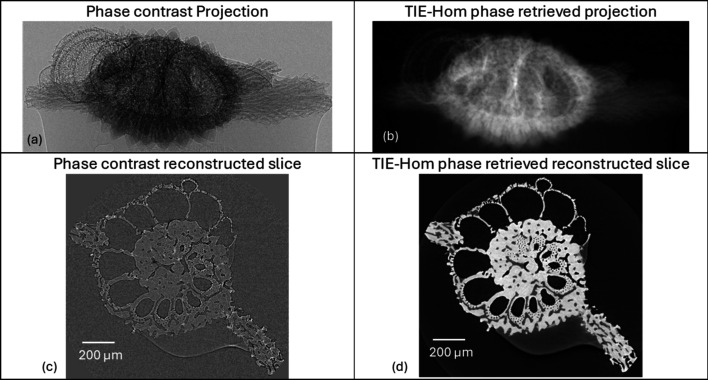
Images of a marine organism (*i.e.* tropical benthic foraminifera from One Tree Island, Great Barrier Reef) showing (*a*) a phase contrast projection after raw correction without pre-processing stage, (*b*) a phase-retrieved projection after raw correction and pre-processing, (*c*) a phase contrast reconstructed slice, and (*d*) a phase-retrieved reconstructed slice. Sample courtesy of Claire Reymond (University of Sydney, Australia).

Applying phase retrieval helps minimize or eliminate bright and dark fringes caused by phase contrast effects, which can interfere with further analysis—particularly segmentation. These fringes often result in poorly segmented sample boundaries. Additionally, the phase retrieval algorithm enhances contrast for regions with slight density differences, improving overall image clarity (Gureyev *et al.*, 2013 ▸). Fig. 6 ▸ shows the phase contrast and the corresponding phase retrieval image of a marine organism in both the projection and reconstructed slice. Phase contrast fringes, appearing as dark and bright fringes around the edges of the sample in the projection image as shown in Fig. 6 ▸(*a*), are corrected through the phase retrieval process, as seen in Fig. 6 ▸(*b*). Phase retrieval is particularly beneficial for imaging low-density objects.

*Beam hardening correction* is another feature in the pre-processing stage, specifically designed to address artefacts caused by dense samples imaged using the white-beam mode. These artefacts occur because lower-energy X-rays in a polychromatic (white) beam are more heavily attenuated than higher-energy X-rays, resulting in a non-uniform linear attenuation coefficient. The correction is applied using the following equation (Zou *et al.*, 2011 ▸),
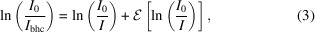
where *I*_bhc_ is the beam-hardening corrected intensity, and 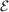 is a correction function defined as the polynomial expansion,
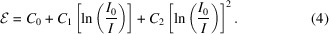
The second-order polynomial fit 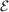 = 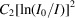 is used to correct for non-linearity in the linear attenuation coefficient, effectively reducing beam hardening artefacts. The parameter *C*_2_ can be iteratively adjusted until the beam hardening artefact is sufficiently minimized.

### 3.3. Tomographic reconstruction

The final stage of the MCT processing pipeline is 3D reconstruction (see Fig. 3 ▸). A critical component of this stage is the *Centre of rotation* (COR) correction, which is essential for achieving a good reconstruction result. The challenge arises because, in practice, the centre of the detector column often does not perfectly align with the rotation axis of the sample stage. Any slight distance between the detector column’s centre and the rotation axis in the projection images must be corrected using the COR feature.

The COR can be determined automatically or specified manually. For the automated method, a Fourier phase correlation-based implementation has been adopted. For this method, a definable sub-region is analysed between a pair of 180° separated projections for the horizontal position of maximum correlation, corresponding to the COR. By default, a central window of the projection’s full width and 25% of its height is used. For particularly homogeneous, or uniform, samples it is sometimes necessary to tune the correlation window width, height and vertical position within the projection to focus on localized features from which an accurate COR can be computed.

**Figure 7:**
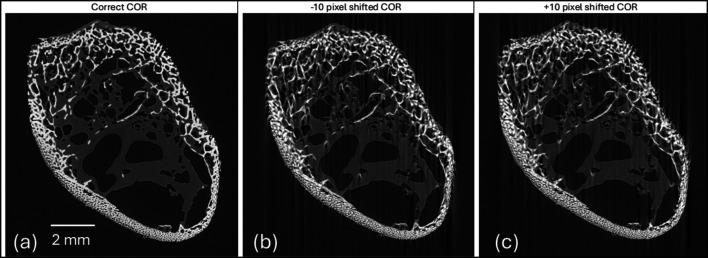
Reconstructed slices of a chicken bone sample after tomography reconstruction, (*a*) using the correct centre of rotation (COR), (*b*) with the COR shifted ten pixels to the left, and (*c*) with the COR shifted ten pixels to the right.

If a COR is successfully computed, the entire projection set is subsequently horizontally transformed, ensuring that the centre of the reconstructed volume consistently aligns with the COR position. Fig. 7 ▸ shows reconstructed slices of a chicken bone sample under three different COR conditions. Shifting the COR negatively or positively produces artefacts in opposite directions as shown in Figs. 7 ▸(*b*) and 7(*c*).

**Figure 8:**
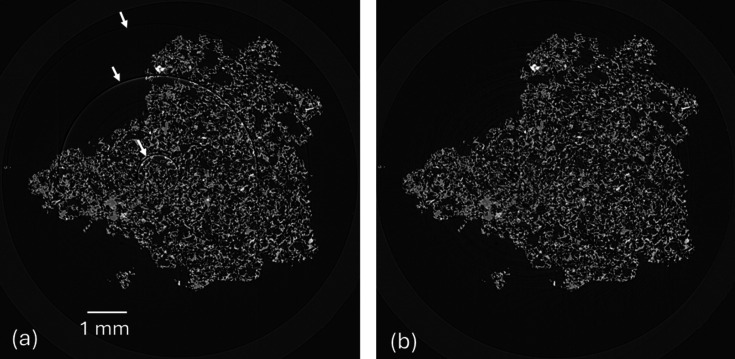
Reconstructed slice of a pumice sample after tomography reconstruction stage, (*a*) before ring artefact correction (RAC) and (*b*) after RAC. White arrows in (*a*) indicate the ring artefacts.

*Ring artefact correction* (RAC) is essential for achieving high-quality reconstructions, as defects on the scintillator or detector system can easily cause ring artefacts. These artefacts typically appear as stripes in the sinogram domain and as rings in the reconstructed image (Titarenko *et al.*, 2010 ▸). They are usually caused by non-ideal normalization affecting specific pixels in the projection images across a range of angles, often due to defects on the scintillator screen or issues with the detector, such as bad pixels or non-linear pixel response. The MCT processing pipeline includes RAC based on sinogram correction methods developed by Nghia Vo (Vo *et al.*, 2018 ▸). Currently, two methods, ‘stripe-based sorting’ and ‘large stripe removal’, are fully implemented, with a third method, ‘dead stripe removal’ nearing completion. When RAC is enabled during the reconstruction stage, the algorithms are applied sequentially for optimal correction. The MCT versions of these algorithms have been re-written and fully GPU-accelerated using native CUDA (Vingelmann & Fitzek, 2020 ▸) and NPP (https://docs.nvidia.com/cuda/npp/index.html) and integrated directly into the pipeline, resulting in a significant increase in performance compared with the original Python-based versions. Most of the gain is achieved from GPU optimized median filtering and the novel hybrid CPU/GPU sorting routine employed for the sort based algorithms. Reconstructed slices before and after RAC can be seen in Fig. 8 ▸, demonstrating the effectiveness of the algorithms in correcting the ring artefacts.

There are 17 filters available for use as the *reconstruction filter* in the MCT processing pipeline, including popular options such as Ram–Lak, Shepp–Logan, Cosine, Hamming and Hann. Each filter offers distinct characteristics that can significantly influence the quality of the reconstructed images. For instance, the Ram–Lak filter is known for its sharp edge enhancement, making it suitable for high-contrast structures, while the Shepp–Logan filter applies a smoothing effect, reducing high-frequency noise. Users can select the most appropriate filter based on the specific requirements of their imaging task, allowing for optimized reconstruction quality across various applications.

The *recon circle filter* is a feature that applies a circular mask to the reconstructed slice, setting all values outside the circle to zero. The circle filter ratio determines the size of this mask, with a value of 1 meaning the circle’s diameter matches the full width of the reconstructed image. The default setting of 0.98 reduces the diameter to 98% of the image width, effectively trimming 2% from the original.

The MCT processing pipeline supports *limited-angle tomography*, also known as tomosynthesis—an alternative 3D reconstruction technique designed for imaging large, flat and laterally extended objects (Rantala *et al.*, 2006 ▸). In tomosynthesis, projection images are acquired and processed over a limited angular range (typically around 100° or less) to prevent near-zero transmission through the thickest part of the sample during rotation. The pipeline utilizes the standard FDK/FBP algorithms from *ASTRA Toolbox* (van Aarle *et al.*, 2016 ▸), which can directly reconstruct the specified range of projection angles provided with full 3D multi-GPU implementations.

The FDK algorithm (Vaniqui *et al.*, 2019 ▸) is the default algorithm employed for CT reconstruction in the MCT pipeline due to the very slight cone-beam like properties of the beam and geometry. The cone beam algorithm delivers higher accuracy results (Stevenson *et al.*, 2012 ▸). In addition, filtered back projection (FBP), and the iterative CT reconstruction algorithms, simultaneous iterative reconstruction technique (SIRT) (Wei *et al.*, 2013 ▸) and conjugate gradient least squares (CGLS) (Soleimani & Pengpen, 2015 ▸), with full 3D GPU implementations from the *ASTRA Toolbox* library are available. These algorithms are not directly exposed for users in the GUI; however, they can be used via the custom defined pipelines in MCT Python *Jupyter Notebooks*.

## 4. Performance assessment

**Figure 9:**
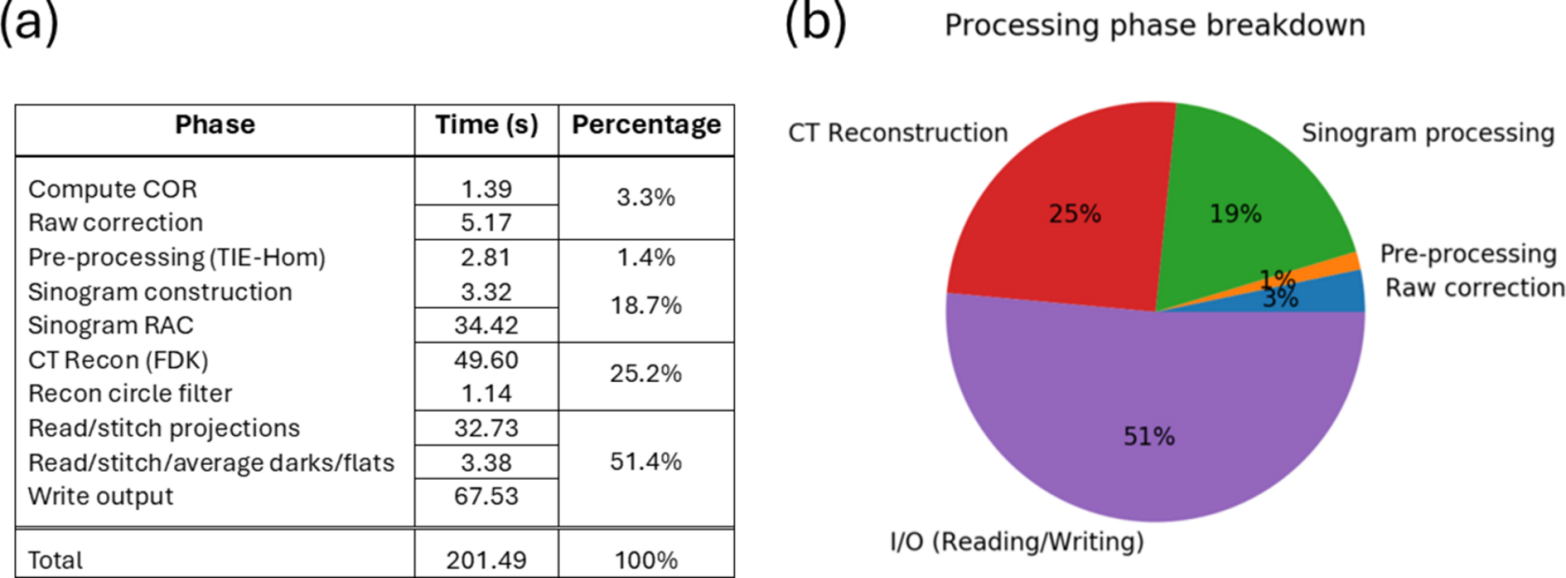
Breakdown of processing phases by execution time of a single full-field acquisition dataset. (*a*) Detailed values and (*b*) percentage distribution.

To assess and characterize performance, the MCT reconstruction pipeline was benchmarked by directly measuring individual phases within the pipeline. Fig. 9 ▸(*a*) displays the breakdown of measured execution times for the most common processing scenario, being the CT reconstruction of a single (non-stitched) full-field acquisition dataset.

Such a dataset generally consists of ∼1820, 2560×2160 16-bit input projections (∼21 GB). When reconstructed, a 2560×2560×2160 32-bit floating-point image volume (∼53 GB) is produced. Fig. 9 ▸(*b*) illustrates the relative proportion of execution time of the phases grouped into five main categories: raw-correction, pre-processing, sinogram processing, CT reconstruction and I/O. As can be seen, I/O, both reading and writing, is the dominant component of the ∼200 s total execution time, consuming just over 50% of the total time to read and write almost 74 GB of data. As to be expected, the CT reconstruction phase is the next most time-consuming, contributing around 25% of the total time, with RAC not far behind with just less than 20%. In comparison, the remaining computational phases, such as raw correction and pre-processing, are relatively insignificant to the overall processing time.

**Figure 10:**
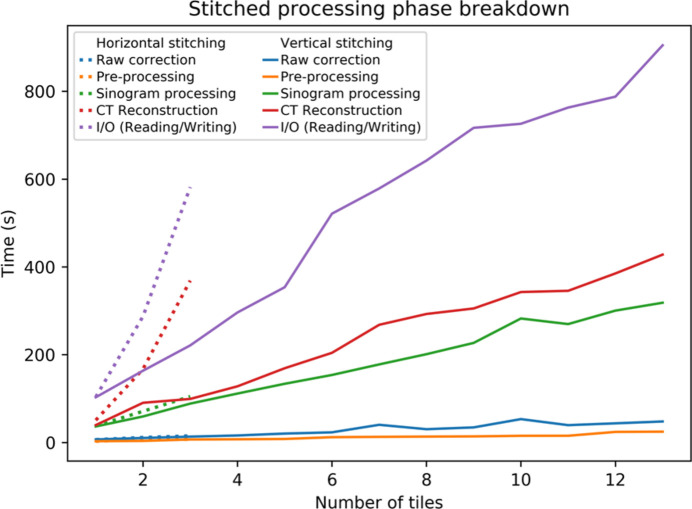
Breakdown of processing phase execution times as a function of the number of tiles, for both horizontal stitching (dotted line) and vertical stitching (solid line).

When processing stitched datasets, the computational performance profile diverges markedly depending on whether horizontal and/or vertical stitching is employed. For CT reconstruction algorithms, computational load is directly proportional to the width of the projections raised to an integer power. As such, increasing the width of input projections due to horizontal stitching has a dramatic effect on the overall processing time. Moreover, an increase in projection width also results in a corresponding increase in both the width and depth of the reconstructed volume, thus increasing the magnitude of the output file. Fig. 10 ▸ illustrates the computational impact of horizontal versus vertical stitching for a specified number of tiles accumulated over the five processing categories. Note that for horizontal stitching the sampling requirement of π/2 for the number of projections must be satisfied.

As before, each ‘tile’ is a separate full-field acquisition of 1820, 2560×2160 16-bit projections. The solid line plots in Fig. 10 ▸ show times for processing 1 up to 13 vertical tiles. Processing times generally scale linearly across all categories as the number of vertical tiles increases. Moreover, as in the non-stitched case, a consistent ∼50% proportion of the total time is spent performing I/O. At the top end, the 13-tile vertically stitched dataset takes almost 30 minutes in total to process and produces a 2564×2564×18817 32-bit floating-point, 461 GB reconstructed volume. It should be noted that the number of vertical stitches is sometimes determined by the vertical beam roll-off, which can be a limiting factor for the FOV.

The dotted lines in Fig. 10 ▸ display times for processing between 1 and 3 horizontally stitched tiles, and, in comparison with the vertical stitching, times for the I/O and CT reconstruction phases increase dramatically and non-linearly due to the increased stitched projection width and the corresponding increase in both CT reconstruction computation and output volume size. The 3-tile horizontally stitched dataset takes just under 18 minutes in total to process and produces a 7123×7123×2162 32-bit floating-point, ∼409 GB reconstructed volume. Due to the rapid increase in both file size and computational times we encourage MCT users to limit the use of horizontal stitching where possible.

At the time of writing, the largest stitched dataset processed during beam time on MCT was a 5×4 tiled acquisition resulting in a ∼3 TB, 10387×10387×7401 reconstructed volume. A primary design goal of the MCT processing pipeline was to allow acquired data to be processed rapidly during beam time, ideally such that processing time is approximately equivalent to acquisition time, thereby permitting processing to be interleaved with acquisition. Additionally, rapid processing capabilities can be leveraged to guide experimental decisions and optimize parameters.

As the results show, I/O operations—particularly reading large projection datasets from storage and writing reconstructed volumes—consume a significant proportion of the total run time, generally around 50%. In contrast, raw correction and pre-processing steps such as phase retrieval and ring artefact correction benefit substantially from GPU acceleration. The in-house-developed CUDA-based implementations provide significant speedups, often by an order of magnitude compared with CPU-based versions, making previously time-consuming operations feasible within experimental timeframes. The pre-processing stage uses a native CUDA implementation, offering acceleration of approximately a 10× speedup compared to a CPU version. For example, on a large 5×4 stitched dataset (4087 × 2366 × 1831), the TIE-Hom phase-retrieval process (originally one of the longest phases of processing) has been reduced from ∼373 s to ∼21 s on a single-GPU machine (‘MCT Offline’). For this specific phase, each projection image requires the computation of a forward Fast Fourier transform (FFT) (padded to the next power of two), multiplication by Fourier filter followed by an inverse FFT. On the high-performance node with three GPUs (‘MCT Online’) the stage scales linearly giving an additional 3× speedup. The quoted 10× speedup relates specifically to the current native multi-GPU (CUDA) implementation for the phase-retrieval (TIE-Hom) step. Firstly, all code in the MCT pipeline is implemented in C++ and all computationally intensive parts are multi-threaded. Also worth noting is that the Intel TBB library is used for the threading back-end which offers generally better multi-threaded performance. In this specific case, the original CPU-only implementation used the FFTW library to compute padded forward and inverse 2D FFTs for each projection and then applied multi-threaded TIE-Hom Fourier filtering. The gains achieved by the CUDA GPU implementation can be primarily attributed to the use of optimized ‘batched 2D’ FFT computations via cuFFT over multiple-GPU’s combined with the asynchronous kernel launches of the Fourier kernel calculation. This leads to a very efficient streamed pipeline with minimal latency between the computation of multiple projections.

Finally, 3D reconstruction, while computationally intensive, also scales efficiently with GPU resources, particularly when streaming CT techniques are applied to very large, tiled datasets. Reconstructing large datasets—for example, a 6× horizontal stitching—can result in reconstructed volumes of approximately 12K × 12K × 2K in size and a reconstruction volume of about 1.1 TB. To handle such large-scale reconstructions, a *streaming CT* approach is required. For the streaming CT method, sufficient memory is needed to hold all projections in memory, primarily to avoid additional processing passes and the need for temporary I/O. However, the reconstruction code is designed to utilize currently available memory. If sufficient memory to hold the full reconstruction volume is unavailable, the system automatically splits the process into smaller vertical slabs. Each slab is reconstructed using the available memory and then written to an HDF5 file incrementally. This method keeps memory usage manageable and enables the reconstruction of much larger datasets. Importantly, this approach does not significantly increase processing time, as it still operates entirely in memory and performs the same amount of file I/O. The main limitation of the streaming CT approach is that it cannot currently generate a secondary output file, since producing such output requires the entire reconstructed dataset to remain in memory for rescaling, conversion or binning operations.

## 5. Conclusion

The continued development of the MCT beamline at the Australian Synchrotron reflects a strong commitment to supporting user demands for advanced X-ray CT research across a wide range of scientific disciplines. By integrating modern data acquisition systems, flexible user interfaces, high-performance computing infrastructure and scalable storage solutions, the beamline has evolved into a highly adaptable platform capable of meeting diverse experimental needs. These advancements not only enhance data quality and throughput but also improve user accessibility and workflow efficiency. With the inclusion of specialized imaging modalities such as speckle and grid-based techniques, the MCT beamline now supports more complex and application-specific investigations. Collaboration between beamline scientists and the Scientific Computing team ensures that the system is robust, user-friendly and aligned with the needs of the scientific community. As user demand varies and research questions increase in complexity, the MCT beamline is well positioned to deliver high-impact imaging capabilities and drive scientific discovery.

## Figures and Tables

**Table 1:** The three most popular fields of research (FoR) among MCT beamline users on each experiment round for three-years periods

	2022	2023			2024			2025		
FoR	Round 3	Round 1	Round 2	Round 3	Round 1	Round 2	Round 3	Round 1	Round 2	Overall
Most popular	Geology (27%)	Classical physics (12%)	Materials eng. (16%)	Geology (19%)	Materials eng. (15%)	Materials eng. (17%)	Materials eng. (11%)	Geology (10%)	Materials eng. (17%)	Materials eng. (12%)
Second most popular	Evolutionary biology (13%)	Materials eng. (12%)	Geology (10%)	Zoology (9%)	Civil eng. (8%)	Geology (10%)	Classical physics (9%)	Materials eng. (10%)	Ecology (7%)	Geology (9%)
Third most popular	Classical physics (13%)	Medical and biological physics (4%)	Manuf. eng. (6%)	Environ. science and mgmt (6%)	Environ. eng. (5%)	Medical and biological physics (7%)	Resource eng. and extract. metall. (9%)	Climate change impacts and ad. (6%)	Oncol. and carcinogenesis (7%)	Classical physics (5%)

## Data Availability

The data that support the findings of this study are available from the corresponding author upon reasonable request.
